# Correspondence

**Published:** 1895-06

**Authors:** 


### CORRESPONDENCE.

### IMPORTANT CHANGES IN THE PERSONNEL OF THE BUREAU OF ANIMAL INDUSTRY.

The acceptance by Dr. Theobald Smith of the position of Bacteriologist to the Massachusetts Board of Health created a vacancy in the office of Chief Pathologist to the Bureau, which, on the 15th ultimo, was filled by the promotion of Dr. Vieranus A. Moore.

Dr. Smith had been with the Bureau since 1883, and is in part the author of and in part connected with nearly all the publications emanating from the Division of Pathology. Eminently fitted by his complete mastery of modern languages, by his training in Cornell University, Albany Medical School, and Johns Hopkins University, and by his acute perceptions and remarkable memory, and beginning his bacteriological studies at the time of their active development in Germany and France, he has been able to keep pace with the foremost investigators and add his quota of original scientific observations and studies of disease to the general sum of knowledge, thus ranking the Pathological Laboratory among the foremost of the world.

During his term of service the Bureau publications on "hog cholera," "swine plague," "Texas fever," "tuberculosis," "broncho-pneumonia," "glanders," and many others of minor importance have been issued and stand accepted as valuable contributions by the scientific world.

The loss of the experience and able qualities of Dr. Smith to the Bureau has been at a gain to himself, and while, on the one hand, it may be regretted, on the other all men will wish Godspeed in his new position and the hearty enjoyment of the well-earned fifty per cent. increase of salary.

One cannot refrain just here from wondering at the conditions which make the government offices a training-school for preparing young men for service in State, city, or private institutions at such enormous increases of salary over those obtained in the Federal service.

Does it not seem better, inasmuch as the values dealt with and the resources therefrom are practically inexhaustible, and the benefits derived from checking loss in any direction are not to be measured with the costs of investigation, that the government employ the most skilled servants, even though they be compelled to pay professional men a little more than they do clerks in the service an equal time? Until Congress and others in authority do make a change in this respect the government will be used as a training-school and the minor institutions will continue to summon the choice students from it to their aid at a loss and harm to public service. Our civil service has yet much to take in consideration in regard to the selection and retention of men fit for the peculiar duties they are to perform.

The appointment of Dr. Vieranus A. Moore as Chief Pathologist of the Bureau comes in the nature of a well-merited promotion. Dr. Moore has been Assistant Pathologist since 1887, and has not only aided in the detailed work of many of the above-cited publications, but is the author of several independent brochures on bacteriologic investigation and animal diseases. The advancement of Dr. Moore insures that the scientific work of the division will be continued on the same scale of excellence.

While the present chief cannot consummate alone the amount of work that was completed by the former chief and his trained assistant, this will be remedied by the training of other assistants. The restoration of the equilibrium now disturbed by the exchange of a man trained by years of experience for untrained assistants will take time, but will be accomplished.

It is not probable that any radical changes can be made by the Bureau authorities in an endeavor to change from its attitude as a training-school, yet we may hope, as time goes on, that gradual changes will be made looking toward, at least, the retention of the skilled professional labor that it in part creates and knows the value of. It would seem that the best servants should be employed in caring for the enormous live-stock interests of the nation, and are worth more to the Bureau than to any State or institution whose interests are but infinitesimal in importance and worth in comparison with its own.

Yours, etc., X.

---

### THE PEOPLE OF THE STATE OF CALIFORNIA *VS.* DR. R. A. ARCHIBALD.

THOSE who read the April number of the VETERINARY ARCHIVES no doubt noticed a set of resolutions published under the head of "Proceedings of the California State Veterinary Medical Association." These resolutions were drafted and introduced by Dr. R. A. Archibald at a meeting of the California State Veterinary Medical Association held in San Jose in March, 1895, and were adopted by said Association for the purpose of preventing a veterinary surgeon named Thomas Carpenter from gaining the appointment of Meat and Milk Inspector in and for the City of Oakland, California. Shortly after the resolutions were made public Dr. Archibald was arrested and brought before the Police Court of Oakland on a charge of criminal libel, preferred by Dr. Carpenter. The trial that followed was watched with a great deal of interest by the different members of the veterinary profession throughout the State, more especially by the members of the Association, for the reason that the above-mentioned person had on numerous occasions done all in his power to retard the progress of the Association, and the trial was deemed an auspicious occasion to expose the said Dr. Carpenter.

The trial, which came off on the 17th day of May, was bitterly contested from start to finish, but justice eventually prevailed. Much testimony was introduced by the defense regarding the position Dr. Carpenter had taken toward the Association in the past, all of which he positively denied on the witness-stand, although the testimony was given by men whose honesty and integrity is unquestionable; he even denied having anything to do with a scandalous article or protest which was circulated at one of the sessions of the State Legislature against the enactment of a law that would protect the veterinary profession in the State, and which was written over his signature. He stated that his name was placed at the foot of the article referred to without his knowledge or sanction, all of which testimony did not seem to impress the jury, who, after being charged by counsel and judge, and after having retired for fifteen minutes, brought in a verdict for the defense, acquitting Dr. Archibald of the charge of criminal libel.

The result of the case was hailed with delight by the members of the veterinary profession throughout the State, and if what we hear be true the prosecuting witness felt correspondingly chagrined, and rightly so, for seldom has a man had his character exposed as was the character of the prosecuting witness in the above-mentioned case.

It might also be interesting to state that Dr. Carpenter did not gain the position of Meat and Milk Inspector for the City of Oakland, but Dr. F. E. Pierce, who has occupied the position with honor for the past year, has been retained in the office.

---

### UNIFORM VETERINARY TITLE.

My dear Doctor:

Your communication regarding a uniform veterinary title arrived too late for me to get a response in on time. In fact, I only received it the day prior to the date you required it in your office. At any rate, it is a matter to which I have given little thought. I believe the simplest title, one understood by the masses, would be the best, like the French "Veterinaire" or the German "Thierartz." The more complicated names, "D.V.M.," "M.D.V.," "M.R.C.V.S.," "D.V.S.," etc., while well meant, are frequently used in a bigoted sense, which is equivalent to charlatanism. It makes one no better to be an M.R.C.V.S. or M.D.V. than to be simply V.S. or Veterinarian.

The multiplication of titles serves no legitimate purpose, but gives to charlatans, whether possessing diplomas or not, a wider range for juggling with titles. This juggling deceives ignorant people only, but unfortunately these form a large part of our patronage, and the value and health of live-stock is largely in their care.

I consequently hold that the simplest possible title is the best, because it can most quickly be comprehended by the common people. To others our individual ability is the standard by which we are measured, the exact title or even its utter absence counting for nothing. In my own case, as with my fellow-alumni, technically we are without a degree, but it has been the custom to assume one, which has, I believe, been fairly uniform, but no degree is recorded or denoted by the certificate of qualifications.

Beyond the reasons above given I see no need of a uniform degree until we can have uniform education, so that the degree will have a definite value as indicating a certain educational training. As it now stands, some colleges may argue that a big degree issued by them denotes a deeper, more scientific education, but this is an individual quarrel between colleges and does not affect us more than disputed individual knowledge of two veterinarians. A degree can only indicate the alma mater, and the value of the man must at least be measured by the sum of his college training, which includes the competency of college instructors and the individual ability of the man himself.

Very truly yours, W. L. WILLIAMS, V.S.

VIRGINIA CITY, MONTANA, May 29, 1895.

